# Effects of TRPC6 Inactivation on Glomerulosclerosis and Renal Fibrosis in Aging Rats

**DOI:** 10.3390/cells10040856

**Published:** 2021-04-09

**Authors:** Eun Young Kim, Stuart E. Dryer

**Affiliations:** 1 Department of Biology and Biochemistry, University of Houston, Houston, TX 77204, USA; ekim8@central.uh.edu; 2 Department of Biomedical Sciences, University of Houston College of Medicine, Houston, TX 77204, USA

**Keywords:** TRPC6, aging, glomerulosclerosis, tubulointerstitial fibrosis, albuminuria

## Abstract

Canonical transient receptor potential 6 (TRPC6) channels have been implicated in familial and acquired forms of focal and segmental glomerulosclerosis (FSGS) in patients and animal models, as well as in renal fibrosis following ureteral obstruction in mice. Aging also evokes declines in renal function owing to effects on almost every renal compartment in humans and rodents. Here, we have examined the role of TRPC6 in driving inflammation and fibrosis during aging in Sprague-Dawley rats. This was assessed in rats with non-functional TRPC6 channels owing to CRISPR-Cas9 deletion of a portion of the ankyrin repeat domain required for the assembly of functional TRPC6 channels (*Trpc6* ^del/del^ rats). Wild-type littermates (*Trpc6* ^wt/wt^ rats) were used as controls. Animals were evaluated at 2 months and 12 months of age. There was no sign of kidney disease at 2 months of age, regardless of genotype. However, by 12 months of age, all rats examined showed declines in renal function associated with albuminuria, azotemia and increased urine excretion of β2–microglobulin, a marker for proximal tubule pathology. These changes were equally severe in *Trpc6* ^wt/wt^ and *Trpc6* ^del/del^ rats. We also observed age-related increases in renal cortical expression of markers of fibrosis (α-smooth muscle actin and vimentin) and inflammation (NLRP3 and pro-IL−1β), and there was no detectable protective effect of TRPC6 inactivation. Tubulointerstitial fibrosis assessed from histology also appeared equally severe in *Trpc6* ^wt/wt^ and *Trpc6* ^del/del^ rats. By contrast, glomerular pathology, blindly scored from histological sections, suggested a significant protective effect of TRPC6 inactivation, but only within the glomerular compartment.

## 1. Introduction

Aging is associated with structural and physiological changes in the kidney, including progressive declines in glomerular filtration rate (GFR) and renal blood flow (RBF) [1]. Glomerulosclerosis was observed in autopsy samples of more than 70% of people over 40 years of age, with greater prevalence and incidence increasing monotonically thereafter [2]. Glomerulosclerosis in aging is accompanied by increases in urine protein and albumin excretion [3] and an average loss of nearly 10% of GFR and RBF occurs with each decade of life after age 40 [4]. Aging is associated with glomerular enlargement, widening of the glomerular basement membrane, mesangial expansion and podocytopenia [5]. This pattern also occurs in rats [6], although the severity of the changes is strongly dependent on the genetic strain and various external factors, especially diet [7]. Renal senescence is also associated with tubulointerstitial fibrosis and increases in pro-inflammatory signaling molecules [8].

TRPC6 channels have been implicated in the pathogenesis of kidney diseases, especially in familial nephrosis associated with mutations in the *TRPC6* gene [9]. More recent evidence has suggested dysregulation of wild-type TRPC6 channels in acquired glomerular diseases [10] and in in vitro models in which podocytes are exposed to serum or plasma samples from patients with recurrent focal and segmental glomerulosclerosis (FSGS) or factors implicated in the pathogenesis of primary FSGS [11,12]. In addition, genetic inactivation of TRPC6 channels in rats confers partial protection in chronic puromycin aminonucleoside (PAN) nephrosis in rats [13].

TRPC6 knockout in mice reduces tubulointerstitial fibrosis following ureteral obstruction [14], and soluble klotho appears to exert part of its protective effects in vivo by suppressing TRPC6 expression in the kidney [14]. TRPC6 is involved in Ca^2 +^ responses associated with immune cell function [15,16]. Therefore, the effects of *Trpc6* knockout on fibrosis could reflect at least some suppression of innate immunity. On the other hand, TRPC6 inactivation resulted in reduced glomerulosclerosis in an autoimmune model of rapidly progressing glomerulonephritis in rats but did not reduce renal cortical inflammation or tubulointerstitial fibrosis in that model [17]. These observations suggest potential species differences in analyses of TRPC6 function and disease mechanisms.

In the present study, we have examined the contribution of TRPC6 channels to declines in renal function during aging in Sprague-Dawley rats. We have utilized rats in which a portion of the TRPC6 channel essential for function was removed using CRISPR/Cas9 technology, resulting in a *Trpc6* ^del^ allele, as described previously [13]. We report here that *Trpc6* ^del/del^ rats and their wild-type *Trpc6* ^wt/wt^ littermates showed similar declines in renal function by 12 months of age. Both strains showed comparable increases in urine albumin excretion and tubulointerstitial disease as assessed by both histological and biochemical markers. However, there was significantly less glomerulosclerosis in the *Trpc6* ^del/del^ rats compared to their *Trpc6* ^wt/wt^ littermates. Thus, in the rat, TRPC6 inactivation is partially glomeruloprotective, but this was not sufficient to preserve several of the most important aspects of renal function during aging.

## 2. Materials and Methods

### 2.1. Animals

All procedures used in animal experiments were approved by the University of Houston Institutional Animal Care and Use Committee, following NIH and ARRIVE guidelines (PROTO201800033, approved on June 26, 2018). The Sprague-Dawley *Trpc6* ^wt/wt^ and *Trpc6* ^del/del^ rats used in these experiments are described in detail previously [13]. Briefly, CRISPR/Cas9 methods were used to introduce a 239-bp deletion within Exon 2 of *Trpc6* (hereafter referred to as the *Trpc6* ^del^ allele). These procedures were carried out by a commercial vendor (Transposagen Biopharmaceuticals Inc., Lexington, KY, USA). The guide RNAs used for gene editing were designed so as to not target genes encoding closely related TRPC channel proteins [13]. As with all experiments using CRISPR/Cas9 methods, we cannot exclude that there may have been off-target effects elsewhere in the genome. We have previously shown that as a result of the gene edit, the *Trpc6* ^del/del^ rats splice out all of Exon 2 during transcription, allowing small amounts of protein to be produced, but resulting in disruption of an ankyrin repeat domain required for functional TRPC6 channels [13]. In electrophysiological experiments, we were unable to detect functional TRPC6 channels in glomerular cells cultured from *Trpc6* ^del/del^ rats, whereas these were readily detected in *Trpc6* ^wt/wt^ animals [13]. Littermates bred from *Trpc6* ^wt/del^ heterozygotes were genotyped using PCR primers that spanned the deleted region of the *Trpc6* gene, as described in detail previously [13]. Littermates with *Trpc6* ^wt/wt^ and *Trpc6* ^del/del^ genotypes were examined at 2 and 12 months of age. The ratio of males to females was the same in each group. Animals were maintained on 12:12 light–dark cycles and allowed free access to food (5053 PicoLab™ Rodent Diet 20, Lab Supply, Inc., Ft. Worth, TX, USA) and water. On this diet, protein accounted for approximately 25% of calories, fat accounted for 13% of calories, and carbohydrates accounted for 62% of calories.

### 2.2. Immunoblot Analysis and Enzyme-Linked Immunosorbent Assays

Immunoblot of the renal cortex was carried out as described previously [13]. Monoclonal antibodies used in this study were: mouse anti-α-smooth muscle actin (α-SMA) clone 1 A4, used at a dilution of 1:1000, from Sigma-Aldrich, St. Louis, MO, USA; mouse anti-vimentin (used at 1:1000) from Dako Inc., Santa Clara, CA, USA; mouse anti-TRPC5 (used at 1:100) from NeuroMAB, Davis, CA, USA; mouse anti-pro-interleukin−1β (used at 1:1000) from Santa Cruz Biotechnology, Santa Cruz, CA, USA. Polyclonal anti-rabbit NLRP3 (used at 1:1000) was from Abcam (Cambridge, MA, USA; anti-TRPC6 (used at 1:1000), and anti-TRPC3 (used at 1:1000) was from Alomone Laboratories (Jerusalem, Israel). All experiments were quantified by densitometry using Image J™ software. Type I procollagen levels in serum were determined using a commercial assay (LSBio, Inc., Seattle, WA, USA) according to the manufacturer’s instructions. β2–microglobulin (β2–MG) levels in the urine were assessed using an assay from ICL Lab (Portland OR, USA). Albumin was measured in 24-h urine samples using a kit from Ethos Biosciences (Philadelphia, PA, USA).

### 2.3. Histopathology and Immunohistochemistry

Portions of kidney from animals in each group were fixed in 10% buffered formalin, embedded in paraffin, and 4-µm sections were stained with periodic acid–Schiff’s (PAS) or Masson’s trichrome methods as described previously [13,17]. Using PAS-stained sections, 25–50 glomeruli from each animal were blindly scored for glomerular injury as described previously [13]. To examine tubulointerstitial injury, for each animal, at least 20 tubulointerstitial fields were examined using microscopes with 20x objectives for the presence of tubular dilatation, tubular atrophy, protein casts, interstitial infiltrates and interstitial fibrosis. Using these criteria, each section was graded as (0) normal; (1) mild change; (2) moderate change; (3) severe change.

### 2.4. Statistical Analyses

All statistical analyses were carried out using public-access computational tools (http://www.vassarstats.net). Throughout, data are presented as mean ± SD and *p* < 0.05 is regarded as significant. Immunoblot assays were analyzed by densitometry. The data are presented as fold changes relative to the lowest value observed in a control group. Data on 24-h urine albumin excretion and other quantitative measures of renal function are presented as mean ± SD from *N* = 7 rats per group. Histological data were quantified with 6 animals per group. Data were analyzed by two-way ANOVA followed by Tukey’s Honest Significant Difference post hoc test. The two independent variables were genotype (*Trpc6* ^wt/wt^ versus *Trpc6* ^del/del^) and age (2 months versus 12 months). A statistically positive result was inferred when *F* values for the interaction between age effects and genotype indicated *p* < 0.05.

## 3. Results

All experiments shown here were carried out on *Trpc6* ^wt/wt^ and *Trpc6* ^del/del^ rats at 2 and 12 months of age. Data here are presented only for 2 and 12 months because a significant of number of the rats in our colony died before reaching 18 months of age, which would result in marked skewing of data. For unknown reasons, our colony of rats appears to show adverse consequences of age substantially earlier than is typical for the Sprague-Dawley background, with marked age-related degeneration apparent by 12 months. This occurred in *Trpc6* ^wt/wt^ and *Trpc6* ^del/del^ rats. It is possible that this is due to an off-target effect of CRISPR/Cas9 gene editing that segregates independently from the deletion in *Trpc6*.

**Figure 1:**
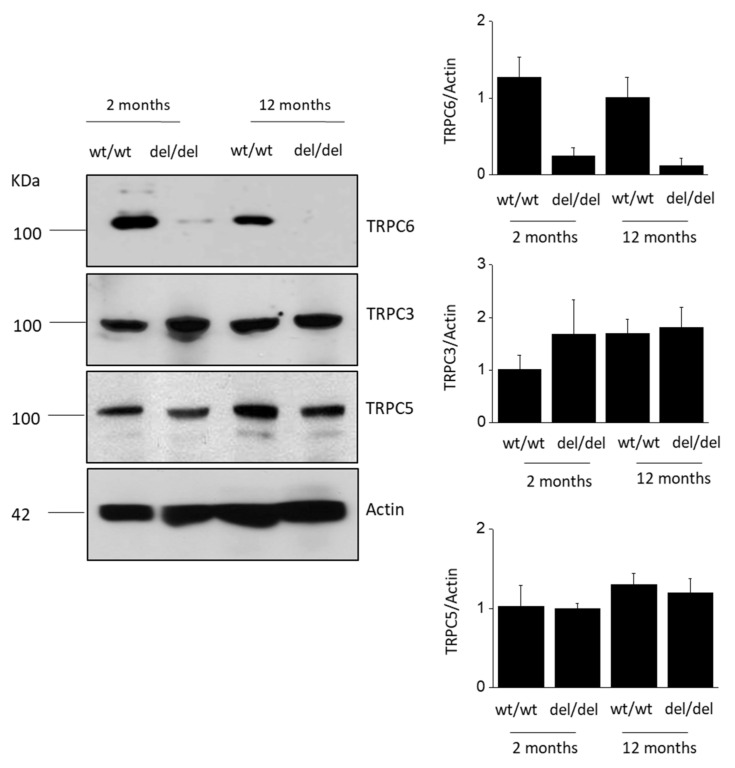
Immunoblot analysis of TRPC6, TRPC3 and TRPC5 abundance from renal cortex of *Trpc6* ^wt/wt^ and *Trpc6* ^del/del^ rats at 2 and 12 months of age as indicated. Representative blots are shown to the left. Bar graphs to the right denote mean ± SD of densitometric analyses with three animals in each group.

TRPC6 is easily detected by immunoblot analysis of the renal cortex from *Trpc6* ^wt/wt^ rats (Figure 1). In addition, a small amount of TRPC6 protein can also be detected by immunoblot analysis in the renal cortex of *Trpc6* ^del/del^ rats (Figure 1), as we described previously [13]. However, *Trpc6* ^del/del^ rats do not express functional TRPC6 channels, which we confirmed in earlier electrophysiological experiments [13]. In the renal cortex of *Trpc6* ^wt/wt^ rats, there does not appear to be a change in total TRPC6 abundance at 12 months compared to 2 months of age (Figure 1). As we also described previously, there is an increase in the total abundance of TRPC3 in the renal cortex in *Trpc6* ^del/del^ rats compared to *Trpc6* ^wt/wt^ littermates at 2 months of age [13]. However, this did not increase further as *Trpc6* ^del/del^ rats aged to 12 months. In the present study, we observed a consistent increase in total TRPC3 abundance at 12 months in *Trpc6* ^wt/wt^ rats compared to at 2 months of age, up to the level seen in *Trpc6* ^del/del^ rats. In our previous electrophysiological studies of *Trpc6* ^del/del^ rats, we found no evidence that TRPC channels could be activated by G-protein-mediated signaling pathways that robustly activate TRPC6 [13]. It is possible that podocyte TRPC3 channels do not traffic to the cell surface in the absence of TRPC6, or in the absence of TRPC6, they require different stimuli to become active. In the present study, we did not observe any significant change in TRPC5 abundance in the renal cortex with increased age in either *Trpc6* ^wt/wt^ or *Trpc6* ^del/del^ rats, and TRPC5 abundance was similar regardless of whether or not functional TRPC6 channels were present; see Figure 1.

**Figure 2:**
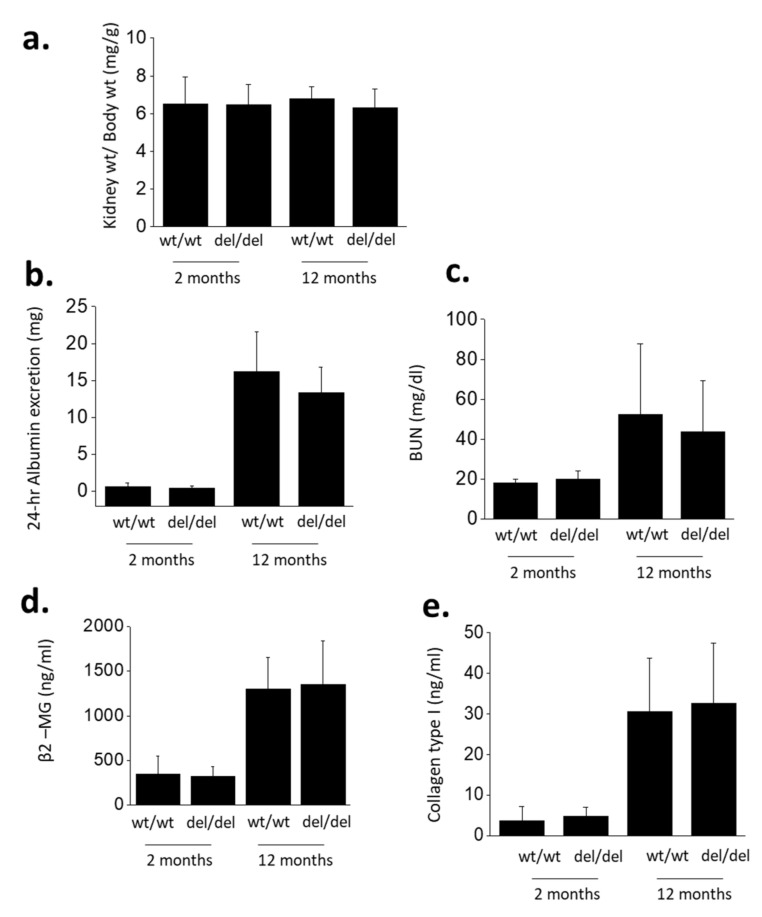
Markers of renal function in *Trpc6* ^wt/wt^ and *Trpc6* ^del/del^ rats at 2 and 12 months of age as indicated. Mean ± SD are shown for kidney weight/body weight (**a**), 24-h urine albumin excretion (**b**), blood urea nitrogen (BUN) (**c**), urine β2-microglobulin (**d**) and serum procollagen type 1 peptide (**e**). Two-way ANOVA of these data indicates that absence of functional TRPC6 channels had no effect on declines in these renal function parameters during aging.

Several measurements revealed marked declines in renal function in these rats at 12 months of age compared to 2 months of age (Figure 2). We did not observe age-related changes in kidney weight/body weight ratios (Figure 2a). However, 12-month-old animals showed increases in 24-h urine albumin excretion (Figure 2b), blood urea nitrogen (BUN) (Figure 2c) and urine β2–microglobulin (β2–MG) (Figure 2d). Recall that β2–MG is a urine biomarker for proximal tubule dysfunction, and that the proximal tubule, especially the S1 segment, plays a major role in recapturing a portion of albumin that escapes through the glomerular filter [18]. The increase in urine albumin excretion seen here is comparable to that reported previously in aging rats [19]. We also observed increases in serum procollagen type 1 peptide, a general marker for fibrotic processes (Figure 2e). Except in the case of kidney weight/body weight ratios, two-way ANOVA of these experiments revealed robust effects of age (*p* < 0.001) but no effect of genotype and no interaction effect between genotype and age. Therefore, the global absence of functional TRPC6 channels did not result in any protection from age-related declines in overall renal function in our colonies.

**Figure 3:**
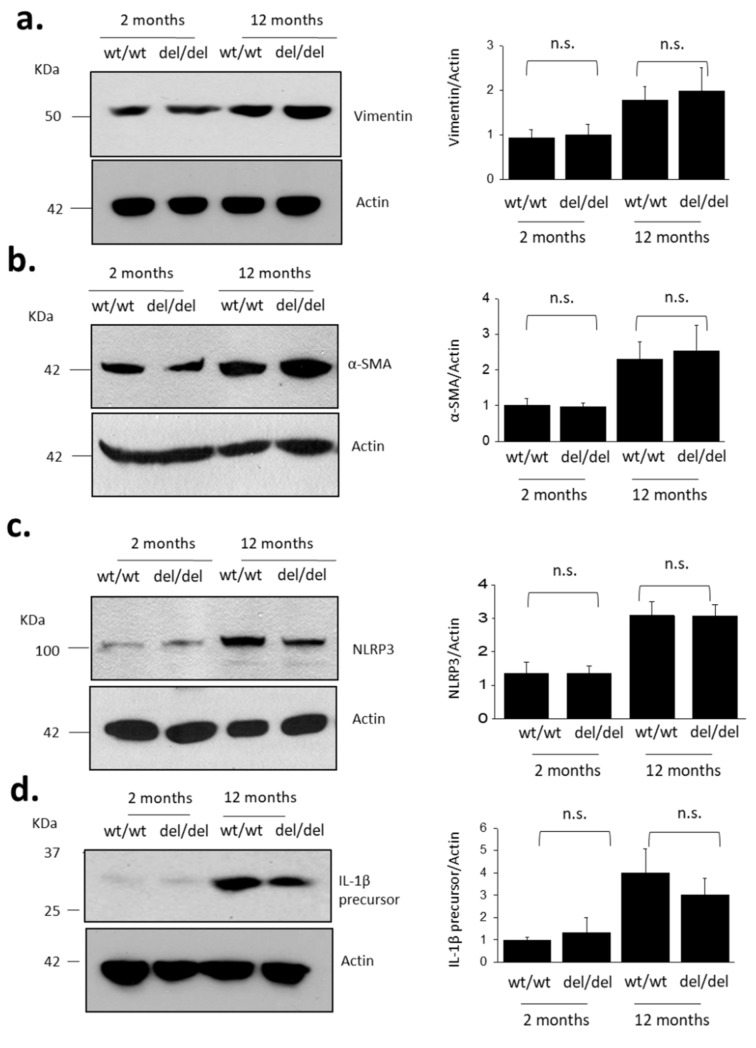
Aging increases abundance of fibrosis and inflammation markers in renal cortex of *Trpc6* ^wt/wt^ and *Trpc6* ^del/del^ rats. Representative immunoblots are shown to the left. Bar graphs to the right denote mean ± SD of densitometric analyses from multiple animals in each group. Data are shown for vimentin (**a**), α-smooth muscle actin (**b**), NLRP3 (**c**) and IL−1β precursor protein (**d**). TRPC6 deletion did not prevent aging-induced increases in markers of renal fibrosis and inflammation. Specific groups were compared by Student’s *t*-test (*n* = 4 animals per group).

Aging also resulted in increased abundance of markers of fibrosis and inflammation in the kidney (Figure 3). Specifically, we observed increases in vimentin (Figure 3a), which suggests increased abundance of mesenchymal cells in the renal cortex, often seen following damage to tubules. We also observed increases in α-smooth muscle actin (SMA) (Figure 3b), which is expected to occur as a result of myofibroblast transdifferentiation [20] and mesangial cell proliferation [21]. Aging was also associated with an increase in the overall abundance of renal cortical Nod-like receptor leucine-rich repeat protein 3 (NLRP3), a major component of inflammasomes [22] (Figure 3c). We also observed increased abundance of pro-IL-1β. This protein is the precursor of pro-inflammatory cytokine IL-1β, which is typically induced during inflammation and cleaved into mature IL-1β by inflammasomes containing NLRP3 [23] (Figure 3d). There was no statistically significant effect of TRPC6 inactivation on the abundance of these disease markers at either 2 or 12 months of age, although there was a trend towards lower IL-1β in *Trpc6* ^del/del^ rats. It is worth noting that podocytes are the major source of IL-1β during glomerulonephritis [24] and a reduction in IL-1β could result in a glomeruloprotective effect.

**Figure 4:**
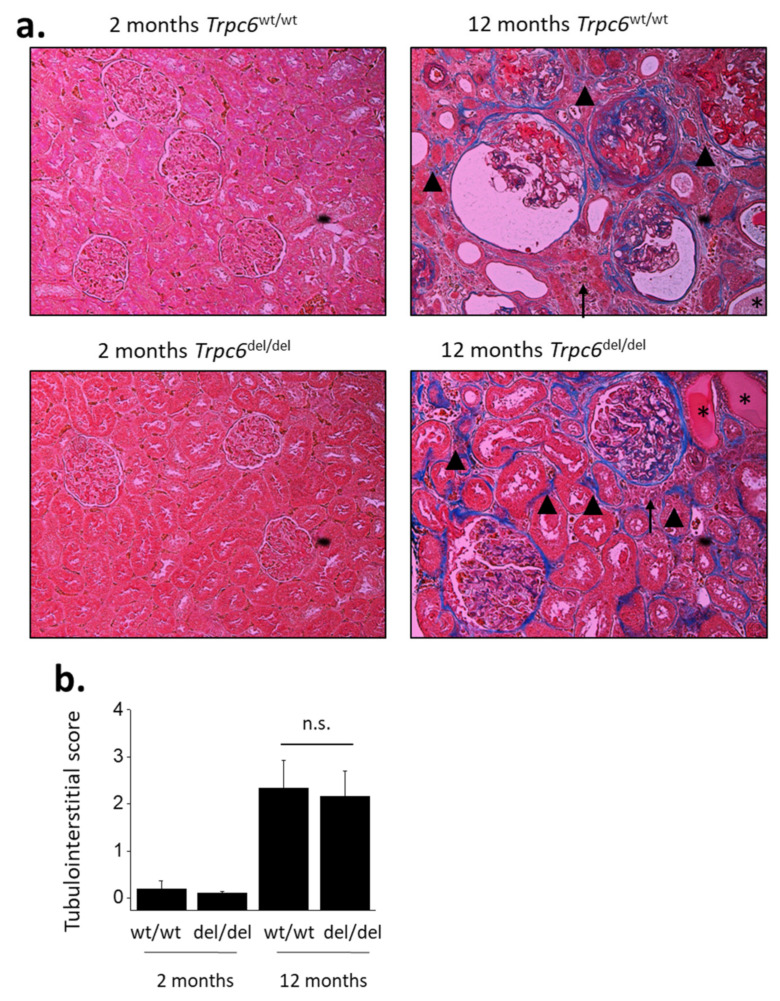
Age-related increases in tubulointerstitial disease. Representative sections stained by Masson’s trichrome method are shown in (**a**). Note extensive collagen (shown in blue) in 12-month-old animals, in glomeruli and in tubulointerstitial areas (arrowheads). Some of the tubules are completely hyalinized (asterisks). Note also areas of cellular infiltration (arrows). These types of images were scored semi-quantitatively by an observer blind to each treatment group for the severity of tubulointerstitial disease (**b**). Two-way ANOVA revealed robust effects of age on tubulointerstitial score (*p* < 0.001) but there was no effect of genotype and no interaction between genotype and age-related changes in this parameter. Data are from 6 animals in each group. Bar graphs show mean ± SD.

**Figure 5:**
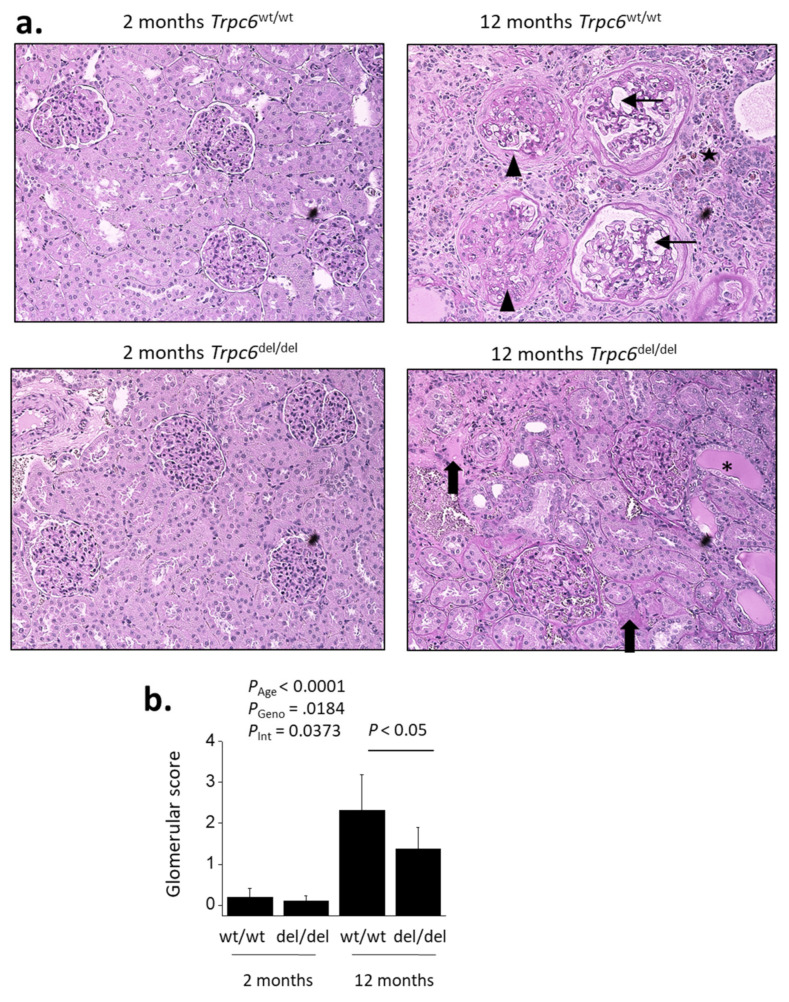
TRPC6 inactivation reduces age-related increases in glomerulosclerosis. Representative sections stained by periodic acid–Schiff’s method are shown in (**a**). Histology of renal cortex is normal in 2-month-old animals regardless of genotype. By contrast, glomerulosclerosis and tubulointerstitial disease could be seen in all 12-month-old animals. Note collapsed and sclerotic glomeruli (arrowheads) and dilated capillary loops (small arrows) in this section from a *Trpc6* ^wt/wt^ rat, as well as regions of cellular infiltration (star). Glomerulosclerosis was less severe and fewer glomeruli were affected in *Trpc6* ^del/del^ animals, as can be seen in the section from the *Trpc6* ^del/del^ rat. Nevertheless, kidney disease was always present regardless of genotype. Note presence of protein casts in tubules (asterisks) and regions of tubulointerstitial fibrosis (large arrow) in the section from the *Trpc6* ^del/del^ rat. These types of images were scored semi-quantitatively by an observer blind to each treatment group for the severity of glomerulosclerosis based on analyses of at least 30 glomeruli from each animal (**b**). These scores reflect only the condition of the glomeruli. Two-way ANOVA revealed robust effects of age on mean glomerular score (*p* < 0.001). There were also significant effects of genotype (*p* = 0.0184) and a significant interaction effect (*p* = 0.0373) between genotype and age-related changes in glomerulosclerosis. Data are from 6 animals in each group. Bar graphs denote mean ± SD.

Age-related changes were also seen in histological analyses using either Masson’s trichrome method (Figure 4) or with PAS staining (Figure 5). Thus, 12-month-old rats exhibited glomerulosclerosis and dilated capillary loops, as well as changes in tubules and in the interstitium. There was a substantial degree of fibrosis seen most clearly by increases in collagen revealed in Masson’s staining (Figure 4), and many tubules contained protein casts clearly seen in PAS staining (Figure 5). There were also indications of cellular infiltration into the interstitium. These characteristics were not seen at 2 months of age.

A semi-quantitative analysis of changes in tubulointerstitial compartments from sections stained with Masson’s trichrome revealed a robust effect of age (*p* < 0.001) but no protective effect of TRPC6 inactivation on changes seen in aging (Figure 4b).

By contrast, a semi-quantitative analysis of glomerulosclerosis using PAS-stained sections carried out by an observer blind to the treatment groups (age or genotype) revealed a partial but significant protective effect of TRPC6 inactivation revealed by two-way ANOVA for the interaction between age and genotype on glomerular score (Figure 5).

## 4. Discussion

Aging often results in a decline in renal function. These changes occur throughout the kidney, and they are exacerbated by other factors, including diet or the presence of diabetes, hypertension or a general inflammatory milieu. TRPC6 contributes to certain glomerular pathologies [9,11,13] as well as to tubulointerstitial fibrosis [14]. Presumably, TRPC6 also has a normal function in rat glomeruli. The purpose of the present study was to assess the contribution of TRPC6 channels to aging-related changes in the rat kidney. We hypothesized that TRPC6 inactivation would reduce aging-related declines in renal function, but we also were open to the possibility that loss of the normal TRPC6 functions might exacerbate aging-related changes in renal function. Contrary to either prediction, we observed here that the absence of functional TRPC6 channels in rats neither ameliorated nor exacerbated declines in overall renal function that occur with age and did not consistently reduce any indices tubulointerstitial fibrosis or inflammation that we measured. However, we did observe a partial protective effect in glomeruli in *Trpc6* ^del/del^ rats compared to *Trpc6* ^wt/wt^ littermates. This is consistent with a previous report that soluble klotho reduces glomerulosclerosis in part by the suppression of TRPC6 expression on the podocyte cell surface [25].

The overall pattern in the present study is similar to what we previously observed in the nephrotoxic serum (NTS) model of rapidly progressing glomerulonephritis [17]. In that model, we detected no effect of the presence or absence of functional TRPC6 on overall renal function or on tubulointerstitial fibrosis, which was severe in that model. However, we observed a partial but significant glomeruloprotective effect of a magnitude similar to that seen in the present study. By contrast, in the chronic PAN nephrosis model, we observed that TRPC6 inactivation reduced declines in overall renal function and produced a glomeruloprotective effect as well as a reduction in tubulointerstitial disease [17]. Finally, in the streptozotocin model of type 1 diabetes, we saw no protective effects of global TRPC6 inactivation anywhere in the kidney [26]. The different outcomes in these models may provide insight as to the role of TRPC6 in these processes, at least in rats. We emphasize, however, that one should not assume that observations in rats will hold true in other species or, indeed, in rats with other genetic backgrounds.

To date, we have observed a glomeruloprotective effect with TRPC6 inactivation in three different chronic disease models (chronic PAN nephrosis, anti-GBM autoimmune glomerulonephritis and aging). In the case of chronic PAN nephrosis, we also observed a reduction in tubulointerstitial disease and preservation of renal function by several different measures. In PAN nephrosis, the initial insult (the toxic effect of the drug) is specific to podocytes. There appears to be a selective uptake of PAN by podocytes [27], and the subsequent metabolism of the drug is accompanied by a massive release of reactive oxygen species that kills some of the podocytes. This initial insult is not affected by presence or absence of TRPC6 [13]. The degree of kidney disease that ultimately develops over a period of weeks is directly related to the number of podocytes initially killed by the drug [28]. The disease then spreads to other compartments once glomeruli are sufficiently compromised—for example, when synechiae form between Bowman’s capsule and capillary tufts [29]. Given that podocyte TRPC6 channels are activated by mechanical stimuli [30], it is possible that TRPC6 contributes to podocyte pathology that occurs as a result of chronic glomerular hyperfiltration—for example, by producing calcium overload in the cells.

Aging and NTS glomerulonephritis are distinctly different from PAN nephrosis in that early pathological processes are not limited to a single cell type but instead occur throughout the kidney and are not a direct downstream consequence of an initial selective insult to podocytes. In the case of the NTS model of autoimmune glomerulonephritis, patrolling immune cells that have reacted with deposits on the GBM leave the glomeruli and trigger pro-fibrotic sequelae in other areas of the kidney and elsewhere [31]. In the case of aging, one expects that most or all of the renal cells will undergo some degree of senescence, and, while podocytes are terminally differentiated cells, there is no evidence to indicate that podocytes are more sensitive to aging than other cell types.

Regardless of where in the kidney the disease processes are initiated, as pathological processes proceed, there will be a decrease in the number of functional nephrons, the rate of which depends on the nature of the model. For example, nephron loss will occur more rapidly in autoimmune glomerulonephritis than in aging. The resulting compensatory hyperfiltration adds an additional stress to the nephrons that remain, especially to podocytes, which may detach in response [29]. The data in this study and those obtained previously [13,17] are consistent with a model in which the absence of functional TRPC6 allows podocytes to withstand this chronic stress more effectively (although, for unknown reasons, this did not occur during diabetes [26]). It is possible that mechanically evoked activation of TRPC6 drives a portion of the glomerular pathology [30]. We should also note that a reduction in glomerulosclerosis in aging *Trpc6* ^del/del^ rats compared to their *Trpc6* ^wt/wt^ littermates did not result in improved urine albumin excretion. This is probably because substantial aging-related proximal tubule defects occurred in both genotypes. Recall that damage to the proximal tubule is by itself sufficient to produce substantial albuminuria, which can approach the nephrotic range [19].

A more robust protective effect of TRPC6 knockout than that observed here was reported in a quite distinct model of renal fibrosis that was examined in mice [14]. Specifically, unilateral ureteral obstruction (UUO) results in rapidly progressing tubulointerstitial fibrosis on the operated side that is significantly reduced in *Trpc6* knockout mice [14]. Moreover, *Trpc6* knockout appears to be equally as effective as a *Trpc6*/*Trpc3* double knockout in mice. In the UUO model, the initial insult occurs primarily to distal tubules, which release a host of cytokines that lead to a massive fibrotic response that can be seen within days. It is possible that the protective effect of *Trpc6* knockout in the UUO model is due to the suppression of myofibroblast transdifferentiation and/or other effects on components of innate immune responses [15,16]. Soluble klotho suppresses TRPC6 expression in podocytes [25] and the suppression of TRPC6 underlies the protective effects of soluble klotho in the UUO model [14]. One can only speculate as to why a similar response does not occur in the aging rat or in rats with autoimmune glomerulonephritis [17]. There may be some unique aspect of the UUO model that makes changes in tubulointerstitial compartments more sensitive to TRPC6 knockout. The collecting duct is especially sensitive to UUO, and it is possible that the protective effects of TRPC6 knockout in the UUO model reflect expression of these channels in the collecting duct [32], or in the secretion of cytokines from tubules [33]. Finally, it is possible that compensatory increases in TRPC3 that we observe in *Trpc6* ^del/del^ rats result in fewer effects on cells that contribute to inflammation and fibrosis in this species compared to mice.

In summary, we have shown that global TRPC6 inactivation in rats results in a histologically discernible reduction in glomerulosclerosis in aging animals. In spite of this, TRPC6 inactivation neither prevented nor exacerbated progressive declines in overall renal function and did not prevent the age-related damage and fibrosis seen in other renal compartments or age-related albuminuria. Inhibition of TRPC6 may be therapeutically useful in some glomerular diseases, but the overall effects of TRPC6 inactivation or knockout appear to be disease model-dependent and are probably also strain- and species-dependent.
